# TRAIL Enhances Apoptosis of Human Hepatocellular Carcinoma Cells Sensitized by Hepatitis C Virus Infection: Therapeutic Implications

**DOI:** 10.1371/journal.pone.0098171

**Published:** 2014-06-13

**Authors:** Jae Young Jang, Seong-Jun Kim, Eun Kyung Cho, Soung Won Jeong, Eui Ju Park, Woong Cheul Lee, Sae Hwan Lee, Sang Gyune Kim, Young Seok Kim, Hong Soo Kim, Boo Sung Kim, Wenyu Lin, Raymond T. Chung

**Affiliations:** 1 Institute for Digestive Research, Digestive Disease Center, Department of Internal Medicine, College of Medicine, Soonchunhyang University, Seoul, South Korea; 2 Department of Internal Medicine, College of Medicine, Soonchunhyang University, Cheonan, South Korea; 3 Department of Internal Medicine, College of Medicine, Soonchunhyang University, Bucheon, South Korea; 4 Division of Infectious Diseases, Department of Medicine, University of California, San Diego, La Jolla, California, United States of America; 5 Gastrointestinal Unit, Department of Medicine, Massachusetts General Hospital, Harvard Medical School, Boston, Massachusetts, United States of America; University of Washington, United States of America

## Abstract

Hepatitis C virus (HCV) infection causes chronic liver diseases leading to hepatocellular carcinoma (HCC) and liver failure. We have previously shown that HCV sensitizes hepatocytes to mitochondrial apoptosis via the TRAIL death receptors DR4 and DR5. Although TRAIL and its receptors are selective targets for cancer therapy, their potential against HCC with chronic HCV infection has not been explored yet. Here we show that HCV induces DR4/DR5-dependent activation of caspase-8 leading to elevation of apoptotic signaling in infected cells and also present TRAIL effect in HCV-induced apoptotic signaling. HCV induced proteolytic cleavage of caspase-9 by stimulating DR4 and DR5, resulting in subsequent cleavage of caspase-3. Further, HCV-induced proteolytic cleavage in caspase-8, caspase-9, and caspase-3 was enhanced in the presence of recombinant TRAIL. HCV-induced cleavage in caspase-9 and increase in caspase-3/7 activity was completely suppressed by silencing of either DR4 or DR5. Perturbing DR4/DR5-caspase-8 signaling complex by silencing DR4 and DR5 or by chemical inhibitor specific to caspase-8 led to decrease of HCV-induced cleavage of poly(ADP-ribose) polymerase (PARP), a substrate for caspase-3 during apoptosis, indicating the functional role of caspase-8 in HCV-induced apoptotic signaling network. Furthermore, TRAIL enhanced PARP cleavage in apoptotic response induced by HCV infection, indicating the effect of TRAIL for the induction of selective apoptosis of HCC cells infected with HCV. Given the importance of apoptosis in HCC development, our data suggest that HCV-induced DR4 and DR5 may be considered as an attractive target for TRAIL therapy against HCC with chronic HCV infection.

## Introduction

Hepatitis C virus (HCV) infects about 170 million people worldwide [1]–[4]. In the course of persistent infection, inflammation forms the pathogenetic basis of chronic hepatitis that can lead to fibrosis, which can progress to cirrhosis and, eventually, hepatocellular carcinoma [5]. It has been suggested that dysregulation of the apoptotic process in infected cells might be crucial for establishing chronicity, the failure of antiviral treatment, fibrosis progression, and neoplastic transformation [6].

Regulation of apoptosis is complex and mainly triggered by the activation of so-called death receptors [7]–[9]. These receptors include cluster of differentiation 95 (CD95)/Fas, tumor necrosis factor (TNF)-receptor-1, and TNF-related-apoptosis-inducing-ligand receptors (TRAIL-R) 1 and 2. In contrast to TNF-R1 and CD95/Fas, TRAIL-R1 and -R2, also known as death receptors 4 (DR4) and 5 (DR5), induce apoptosis only in infected or transformed tumor cells [10], but not in uninfected healthy hepatocytes *in vivo* [11], [12].

In addition to inducing apoptosis, these death receptors initiate independent intracellular signaling cascades through mitochondrial dysfunction. Mitochondrial dysfunction plays a critical role in integrating death receptor-initiated apoptosis into a final common pathway by activating a caspase-dependent apoptosis [13]–[16]. TRAIL binding causes the formation of death-inducing signaling complex, resulting in the activation of caspase-8. Active caspase-8 can trigger direct activation of caspase-3 and cleavage of Bid, followed by mitochondria-dependent activation of caspase-9 via cytochrome C release and apoptotic protease activating factor-1 (Apaf-1) activation [17]. However, the mechanism by which caspases are activated is still not well defined in hepatitis C virus infection.

We recently demonstrated that co-infection with HCV and HIV stimulates DR4 and DR5 gene expression and subsequently activates caspase-3 signaling pathway, resulting in induction of mitochondrial apoptosis of human hepatocytes [18]. In this study, we demonstrate the significant contribution of caspase-8 activation to HCV-induced DR4/DR5-dependent hepatocyte apoptosis.

## Materials and Methods

### Cell culture and virus

The human hepatocellular carcinoma Huh7.5.1 cells used in this study were grown in high-glucose Dulbecco's Modified Eagle's Medieum (DMEM; Hyclone) supplemented with 10% fetal bovine serum (Hyclone; Thermo Fisher Scientific, Wilmington, Delaware, USA), 100 U/ml penicillin and 0.1 mg/ml streptomycin (Hyclone; Thermo Fisher Scientific, Wilmington, Delaware, USA). Cell culture-derived HCV JFH-1 (HCVcc) used in this study was propagated and prepared, as described previously [18].

### Reagents and antibodies

Recombinant human TRAIL, pan-caspase inhibitor Z-VAD-FMK, caspase-8 specific inhibitor Z-IETD-FMK, and caspase-9 specific inhibitor Z-LEHD-FMK used in this study were purchased from R&D systems (Abingdon). TRAIL and caspase inhibitors were reconstituted according to the manufacturer's instructions. Primary antibodies used in this study include the following: rabbit monoclonal anti-cleaved PARP (Cell signaling Technology); mouse monoclonal anti-DR4 (Abcam); rabbit monoclonal anti-DR5 (Cell signaling Technology); rabbit monoclonal anti-cleaved caspase-3 (Cell signaling Technology); rabbit monoclonal anti-cleaved caspase-8 (Cell signaling Technology); rabbit monoclonal anti-cleaved caspase-9 (Cell signaling Technology); rabbit monoclonal anti-cleaved Bid (Invitrogen); mouse monoclonal anti-HCV core (Thermo Fisher Scientific); mouse monoclonal anti-α-tubulin (Sigma); mouse monoclonal anti-β-actin (Sigma). The secondary antibodies used for Western blot analysis were HRP-conjugated anti-mouse IgG (Santa Cruz Biotechnology) and HRP-conjugated anti-rabbit IgG (Santa Cruz Biotechnology).

### Small interfering RNA transfection

siGENOME non-targeting siRNA pool #1 and ON-TARGETplus SMARTpool small interfering RNA (siRNA) for DR4 (NM_003844) and DR5 (NM_003842) were purchased from Dharmacon. Huh7.5.1 cells were transfected with siRNAs (50 nM) using Lipofectamine RNAiMAX transfection reagent according to the manufacturer's instructions (Invitrogen) prior to HCVcc infection.

### Real-time qRT-PCR

To analyze the expression levels of DR4 and DR5, total cellular RNA was extracted from Huh7.5.1 cells using RNeasy Mini kit (Qiagen) and subsequently, complementary DNA was synthesized by using GeneAmp RNA PCR kit (Applied Biosystems) according to the manufacturer's instructions. Power SYBR Green PCR Master Mix (Applied Biosystems) was used to quantify the cellular RNA levels. The following primer sets were used for RT-PCR: DR4 forward, 5′-CTGAGCAACGCAGACTCGCTGTCCAC; DR4 reverse, 5′-TCAAAGGACACGGCAGAGCCTGTGCCA; DR5 forward, 5′-GGGAGCCGCTCATGAGGAAGTTGG; DR5 reverse, 5′-GGCAAGTCTCTCTCCCAGCGTCTC; GNB2L1 forward, 5′-GACCATCATCATGTGGAAACTGA; GNB2L1 reverse, 5′-CCGTTGTGAGATCCCAGAGG. GNB2L1 was used as an internal control. Real-time qPCR was conducted by using a StepOnePlus Real-Time PCR System (Applied Biosystems).

### Western blot analysis

Cells were resuspended in lysis buffer (50 mM Tris-HCl, 1 mM EDTA, 50 mM NaF, 1 mM Na3VO4, 1% Triton X-100, 150 mM NaCl, and 10 µg/ml aprotinin) supplemented with protease inhibitor cocktail (Roche). Whole cell lysates were subjected to SDS-PAGE, transferred to nitrocellulose membrane (Millipore), and Western blot analyzed with antibodies against the indicated proteins.

### Caspase-3/7 activity assay

Caspase-3/7 activity in HCV-infected cells was measured using the Caspase-Glo 3/7 assay kit according to the manufacturer's instruction (Promega). Briefly, Huh7.5.1 cells transfected with non-targeting (NT) or specific-targeting siRNA pools for DR4 and DR5 were infected with HCVcc. At 3 days post-infection, caspase-3/7 specific luminescence activity was measured using a Synergy 2 Multi-Mode Microplate Reader (BioTek).

### Statistical analysis

Statistical analysis was performed using a paired student's t-test. Results were expressed as means ± standard deviation of at least three sample replicates.

## Results

### HCV infection stimulates DR4 and DR5 gene expression

**Figure 1:**
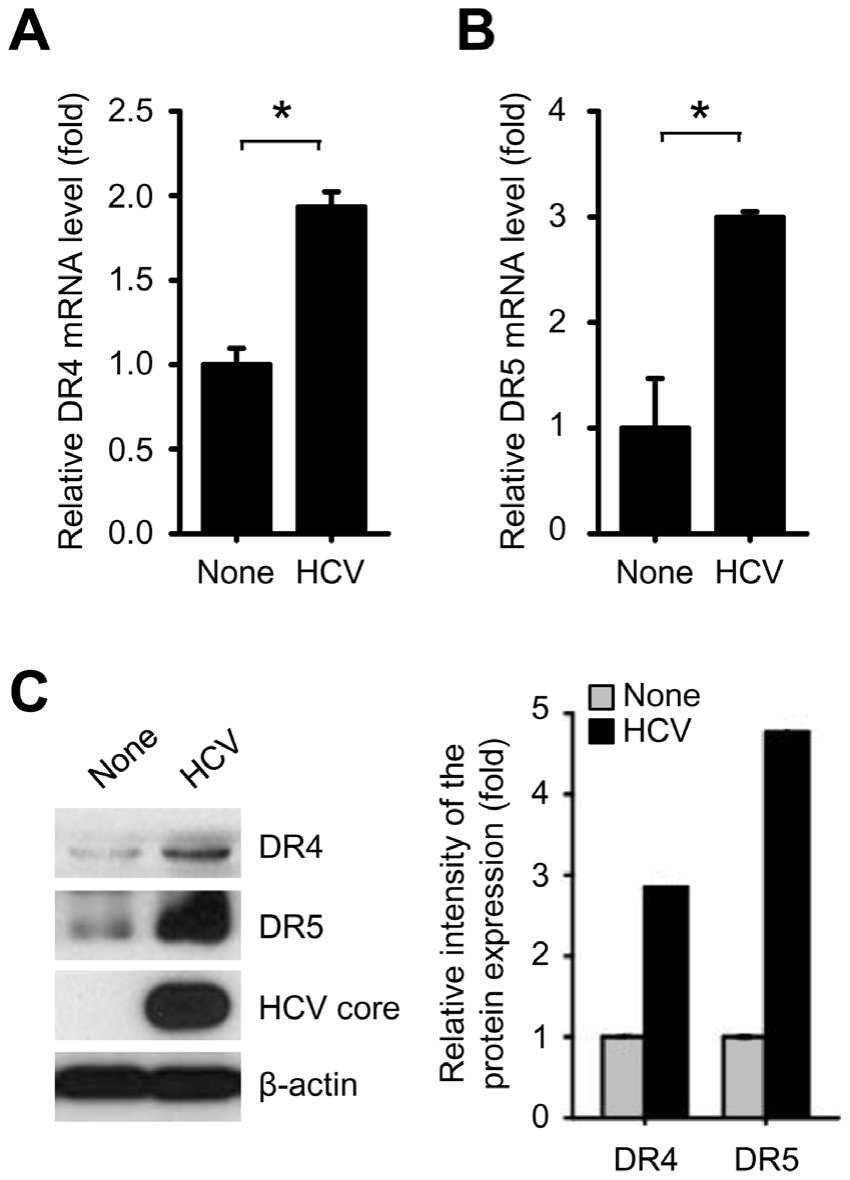
HCV infection stimulates DR4 and DR5 expression. (A-C) Quantitative analyses of DR4 and DR5 gene expression in HCV-infected cells. Huh7.5.1 cells were infected with HCVcc. At 3 days post-infection, cells were used for analysis of DR4 and DR5 gene expression. (A and B) Intracellular mRNA level of DR4 and DR5 genes were analyzed by real-time qRT-PCR. Human GNB2L1 was used to normalize changes in DR4 and DR5 gene expression (mean±SD; n = 3; *p<0.001). (C) The protein expression levels of DR4 and DR5 were analyzed by Western blotting with anti-DR4 and DR5 antibodies. The expression of HCV core protein was analyzed by Western blotting with anti-HCV core antibody. β-actin was used as an internal loading control. The relative intensity of DR4 and DR5 expression normalized to β-actin was analyzed by ImageJ.

We have recently shown that HCV infection activates DR4 and DR5-mediated intrinsic apoptotic signaling in human hepatocytes [18], consistent with other reports [19]–[21]. To evaluate critical determinants on DR4/DR5-dependent apoptotic signaling network induced by HCV infection, we first examined DR4 and DR5 gene expression. As shown in Figs. 1A and B, HCV infection stimulated the level of both DR4 and DR5 mRNA expression, consistent with previous reports [18], [19], and induced consistent increase in DR4 and DR5 protein expression as evidenced by Western blotting with antibodies specific to DR4 and DR5 (Fig. 1C). These results indicate that HCV infection stimulates DR4 and DR5 gene expression to promote DR4/DR5-dependent apoptotic signaling.

### HCV infection activates caspase-8-dependent apoptosis signaling by stimulating DR4 and DR5

**Figure 2:**
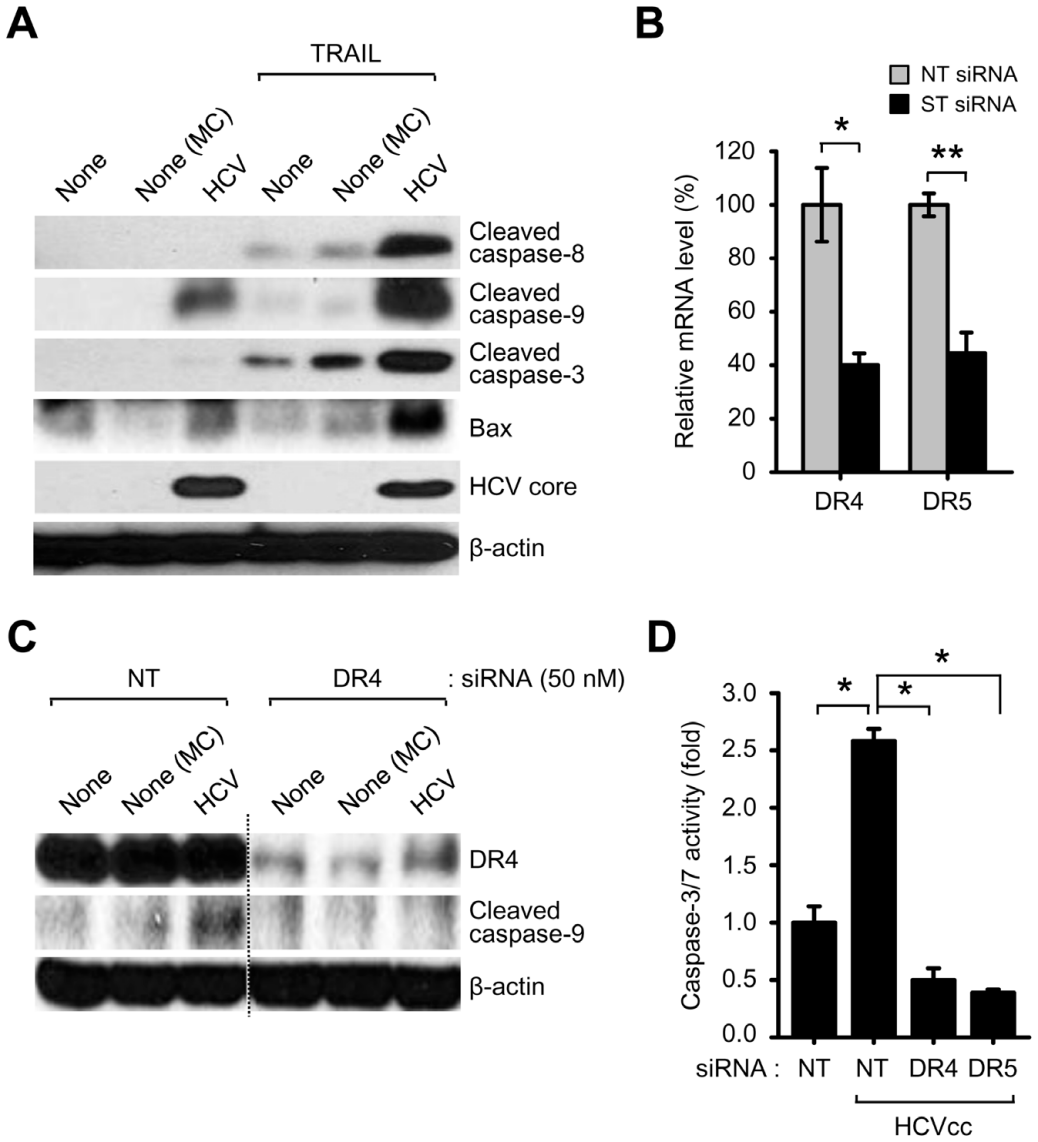
HCV infection activates DR4/DR5-dependent caspase cascade. (A) Western blot analysis of Bax and cleaved caspase-8, caspase-9, and caspase-3 in HCV-infected cells. Huh7.5.1 cells infected with HCVcc in the absence or presence of recombinant TRAIL (100 ng/ml) were used for Western blot analysis with antibodies against the indicated proteins. β-actin was used as an internal loading control. Samples: MC, media control. (B and D) Silencing DR4 and DR5 prevent caspase-9 cleavage and caspase-3/7 activity induced by HCV infection. Huh7.5.1 cells transfected with non-targeting (NT) or gene-specific (ST) siRNA pools targeting DR4 and DR5, respectively, were infected with HCVcc. At 3 days post-infection, the mRNA levels of DR4 and DR5 were analyzed by real-time qRT-PCR (B) (mean±SD; n = 3; *p<0.05, **p<0.01) and whole cell lysates were analyzed by Western blotting with antibodies specific to the indicated proteins (C). (D) The activity of caspase-3/7 was measured according to manufacturer's instructions (mean±SD; n = 3; *p<0.005).

TRAIL induces the recruitment of Fas-associated death domain and procaspase-8 to DR4 and DR5, leading to activation of caspase-8 and subsequent caspase-9/-3-dependent apoptotic signaling pathway [22]–[25]. To investigate caspase-8-mediated apoptotic signaling by stimulating DR4 and DR5 induced by HCV infection, we analyzed whether HCV induces caspase-8 activation leading to subsequent cleavage in caspase-9 and caspase-3 in Huh7.5.1 cells. As shown in Fig. 2A, HCV infection increased the level of cleaved caspase-9 and caspase-3. Furthermore, treatment of HCV-infected Huh7.5.1 cells with recombinant TRAIL elevated the level of cleaved caspase-8, caspase-9 and caspase-3, indicating that TRAIL enhances caspase-8 dependent apoptotic signaling via DR4 and DR5 receptors (Fig. 2A). Consistent with previous results, HCV infection led to Bax activation, a key mediator of apoptosis, in Huh7.5.1 cells (Fig. 2A) [26]. HCV infection also increased caspase-3/7 activity in Huh7.5.1 cells (Fig. 2C). Further, we examined the significance of DR4 and DR5 on apoptotic signaling pathway activated by HCV infection. As shown in Figs. 2C and D, HCV-induced caspase-9 cleavage and increase in caspase-3/7 activity was inhibited by silencing either DR4 or DR5 as evidenced by qRT-PCR (Fig. 2B). Together, these results indicate that caspase-8 activation via DR4 and DR5 is required to promote HCV-induced apoptotic signaling.

### HCV infection elevates DR4/DR5-mediated PARP cleavage via caspase-8 activation

**Figure 3:**
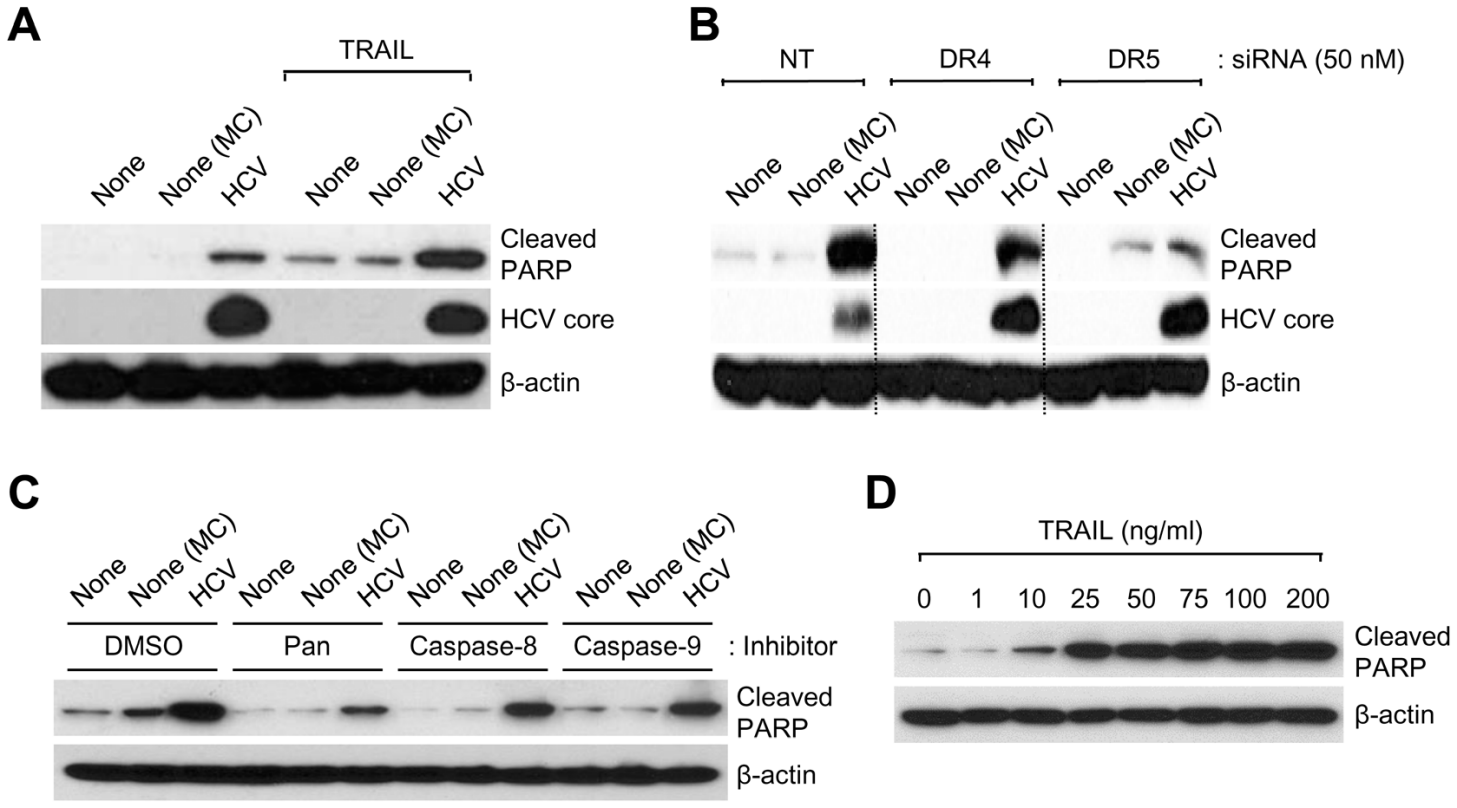
HCV-induced DR4/DR5-mediated caspase-8 activity affects PARP cleavage. (A) Western blot analysis of cleaved PARP in HCV-infected cells. Whole cell lysates extracted from HCV-infected Huh7.5.1 cells grown in the absence or presence of recombinant TRAIL (100 ng/ml) were analyzed by Western blotting with antibodies specific to the indicated proteins. (B) Huh7.5.1 cells transfected with non-targeting (NT) or gene-specific (ST) siRNA pools targeting DR4 and DR5, respectively, were infected with HCVcc. At 3 days post-infection, whole cell lysates were analyzed by Western blotting with antibodies specific to the indicated proteins. (C) HCV-infected Huh7.5.1 cells stimulated with recombinant TRAIL (100 ng/ml) were treated with Z-VAD-FMK (pan inhibitor; 40 µM), Z-IETD-FMK (caspase-8 inhibitor; 40 µM), and Z-LEHD-FMK (caspase-9 inhibitor; 40 µM), respectively, for 72 h before harvest. The expression level of PARP was analyzed by Western blotting with anti-cleaved PARP antibody. (D) Western blot analysis showing dose-dependent effects of recombinant TRAIL on PARP cleavage in HCV-infected cells. HCV-infected Huh7.5.1 cells were treated with recombinant TRAIL for 72 h at the indicated dose points. Whole cell lysates were analyzed by Western blotting with anti-cleaved PARP antibody. Samples: MC, media control. (A-D) β-actin was used as an internal loading control.

**Figure 4:**
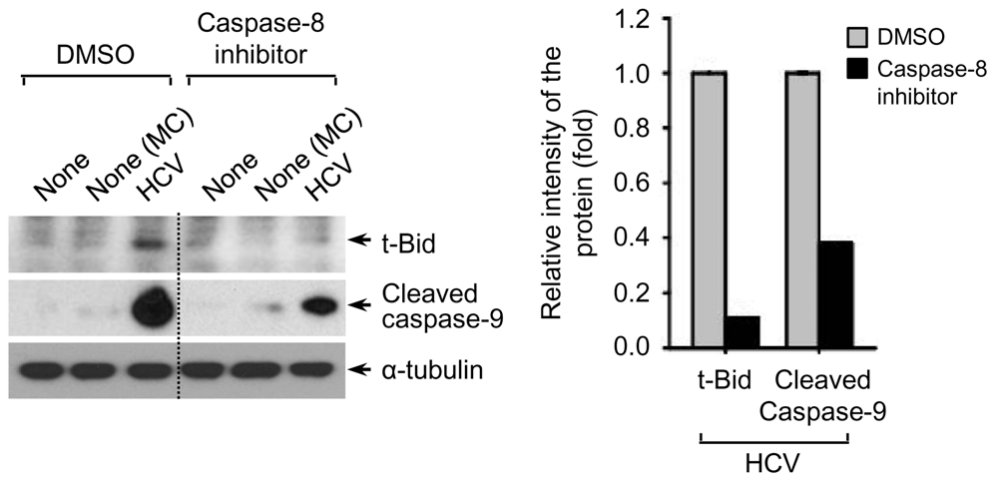
HCV-induced caspase-8 activity modulates caspase-9 cleavage. Huh7.5.1 cells were infected with HCVcc in the absence or presence of caspase-8 inhibitor (20 µM) for 72 h before harvest. The expression levels of truncated Bid (t-Bid) and cleaved caspase-9 were analyzed by Western blotting with antibodies specific to t-Bid and cleaved caspase-9. α-tubulin was used as an internal loading control. The relative intensity of t-Bid and cleaved caspase-9 normalized to α-tubulin was analyzed by ImageJ. Samples: MC, media control.

PARP cleavage by caspase activation is a hallmark of apoptosis [27], [28]. Thus, we investigated proteolytic cleavage of PARP via DR4/DR5-mediated caspase activation in HCV-infected Huh7.5.1 cells. As shown in Fig. 3A, a notable increase in cleaved PARP level was detected in HCV-infected cells compared to uninfected cells (see lane 3). This increase was further augmented by treatment with recombinant TRAIL (Fig. 3A, lane 6), suggesting that TRAIL may induce enhanced apoptosis via DR4 and DR5 receptors of HCV-infected cells. Interference of caspase-8-mediated apoptotic signaling network by silencing either DR4 or DR5 prevented HCV-induced PARP cleavage (Fig. 3B). Next, we examined whether HCV-induced PARP cleavage is mediated by caspase-8 activation using caspase inhibitors specific to caspase-1/3, caspase-8, and caspase-9, respectively. As shown in Fig. 3C, HCV-induced increase in cleaved PARP was suppressed by inhibiting caspase activity. Here, we note that caspase-8 activation, an upstream effector of caspase-9 and caspase-3, mediates DR4/DR5-mediated PARP cleavage in HCV-infected cells. We also analyzed that HCV-induced PARP cleavage is dose-dependently promoted by TRAIL treatment (Fig. 3D). By inhibiting caspase-8 activity, the functional significance of caspase-8 for DR4/DR5-dependent apoptotic signaling in HCV-infected cells was revealed through suppression of HCV-induced Bid cleavage and subsequent caspase-9 cleavage (Fig 4). Caspase-8 activation promotes cleavage of Bid and subsequent translocation of truncated Bid to the mitochondria, eventually resulting in apoptosis via induction of cytochrome C release [17], [29]. Together, these results demonstrate that HCV infection promotes DR4/DR5-mediated PARP cleavage via translocation of caspase 8-truncated Bid.

## Discussion

Apoptosis, or programmed cell death, is a process in which cells activate intracellular death pathways to terminate themselves systematically in response to a wide variety of stimuli [30], [31]. Previous studies have shown that the severity of liver damage in chronic HCV is associated with the degree of hepatocyte apoptosis [32], [33]. However, the contribution of apoptosis or the molecular mechanisms that cause liver cell damage during HCV infection have not yet been clearly defined. Recently, death receptor DR4/DR5 has been recognized as a specific mediator of HCV-induced hepatocyte apoptosis [34]–[36]. Thus, we sought to evaluate critical determinants, particularly caspase-8 activation, of the DR4/DR5-dependent apoptotic signaling pathway induced by HCV infection.

A therapeutic potential of TRAIL for liver cancer with persistent HCV infection has been overlooked. TRAIL receptors including DR4 and DR5 are very attractive targets for cancer therapy [37]. Hence, in this work, our research purpose was (1) to examine the role of caspase-8, one of important determinants of DR4/DR5-mediated apoptosis signaling network during HCV infection as well as (2) to illuminate the possibility of TRAIL use for therapy of hepatocellular carcinoma caused by HCV infection.

It is commonly assumed that virus clearance requires the destruction of infected cells by virus-specific cytotoxic T-lymphocytes via the CD95 receptor, TNF-receptor-1 and TRAIL receptors DR4 and DR5. If death receptor-mediated apoptosis works adequately, it is beneficial for the organism and may lead to elimination of virus-infected cells. However, dysregulation of this system might result in liver damage. Therefore, TRAIL receptors, which selectively kill virus-infected cells while leaving uninfected cells intact, have evolved as a prime physiological mechanism in order to selectively eliminate virus-infected cells [38]–[40]. We and others have previously shown the stimulation of DR4 and DR5 in the liver of patients with chronic HCV as well as in HCV-infected cells [11], [18], [21], [39], [41]. In contrast, few reports have suggested that HCV infection does not affect DR4 or DR5 expression [20], [42]. In this study, we clarify that HCV infection stimulates DR4 and DR5 gene expression at both the transcriptional and translational levels in Huh7.5.1 cells, as evidenced by qRT-PCR and Western blot analysis (Fig. 1). Consistent with our results, Zhu *et al* have shown that HCV infection upregulates DR4 and DR5 expression levels and triggers TRAIL-mediated apoptosis in human hepatocellular carcinoma LH86 cells [21]. They also demonstrated that TRAIL expression is correlated with the number of apoptotic LH86 cells [21].

A number of liver diseases are characterized by elevated caspase activation and apoptosis. Caspase is a cysteine protease which plays a critical role in initiating and executing the apoptotic signaling by degrading several cellular components, resulting in the apoptotic phenotype [43]. In death receptor-mediated apoptosis, CD95L-, TNF- and TRAIL-induced signal transduction leads to the formation of a death-inducing signaling complex which results in the proteolytic processing of caspase-8 and activation of downstream effector caspases such as caspase-3, caspase-6, and caspase-7, known as an extrinsic pathway [38], [44], [45]. The intrinsic apoptotic pathway regulated by the B cell lymphoma 2 (Bcl-2) family proteins results in activation of caspase-9, which in turn activates the downstream effector caspases [43]. Effector caspases are a class of enzymes responsible for the execution of apoptosis and their activation was observed in a considerable percentage of hepatocytes in liver biopsies from chronic HCV infection [46]. Lan *et al* have shown that HCV infection induces TRAIL-mediated mitochondrial apoptosis via caspase-9 activation [20], however, the precise mechanism still remains to be clarified. In Fig. 2, we show that HCV infection induces the activation of caspase-8 and subsequent downstream effectors caspase-9 and caspase-3 in response to TRAIL. The functional significance of DR4 and DR5 in activation of caspase-dependent apoptotic signaling induced by HCV infection was also clarified by reduction of caspase-3/7 activity by DR4 and DR5 silencing (Figs. 2C and D). We propose that HCV infection promotes caspase-8 activity followed by activation of caspase-9 and caspase-3, as evidenced by suppression of DR4/DR5-mediated PARP cleavage via translocation of caspase 8-truncated Bid. Taken together, our data demonstrate the functional significance of DR4/DR5-mediated caspase-8 activation as a critical mediator of HCV-induced apoptosis. Efforts to inhibit the TRAIL apoptotic program may be warranted to retard the progression of HCV-related advanced liver disease.
